# Korean Traditional Medicine in Treating Patients with Mild Cognitive Impairment: A Multicenter Prospective Observational Case Series

**DOI:** 10.1155/2020/4323989

**Published:** 2020-02-22

**Authors:** Yujin Choi, Young-Eun Kim, Ui Min Jerng, Hwan Kim, Sung Ik Lee, Ga-Na Kim, Seung-Hun Cho, Hyung Won Kang, In Chul Jung, Kyungsun Han, Jun-Hwan Lee

**Affiliations:** ^1^Clinical Medicine Division, Korea Institute of Oriental Medicine, 1672 Yuseong-daro, Yuseong-gu, Daejeon 34054, Republic of Korea; ^2^Future Medicine Division, Korea Institute of Oriental Medicine, 1672 Yuseong-daro, Yuseong-gu, Daejeon 34054, Republic of Korea; ^3^Department of Internal Medicine, Sangji University, Wonju, Gangwon 26339, Republic of Korea; ^4^Department of Oriental Neuropsychiatry, Dunsan Korean Medicine Hospital of Daejeon University, 75-0, Daedeok-daero 176 Beon-gil, Seo-gu, Daejeon, Republic of Korea; ^5^Inam Neuroscience Research Center & Department of Neurology, Sanbon Medical Center, Wonkwang University School of Medicine, 321, Sanbon-ro Gunpo City, Gyeonggi–do 15865, Republic of Korea; ^6^Department of Neuropsychiatry, College of Korean Medicine, Kyung-Hee University, 26, Kyungheedae-ro, Dongdaemun-gu, Seoul 02447, Republic of Korea; ^7^Department of Korean Neuropsychiatry Medicine, Wonkwang University, 460 Iksan-daero, Iksan-si, Jeollabuk-do 54538, Republic of Korea; ^8^Korean Medicine Life Science, University of Science & Technology (UST), Campus of Korea Institute of Oriental Medicine, Daejeon 34113, Republic of Korea

## Abstract

In Korea, patients with mild cognitive impairment can choose to receive treatment of Korean medicine, and Korean medicine hospitals provide specialized medical care for the prevention and management of cognitive disorders. The aim of the study is to explore the role of Korean medicine therapy for patients with mild cognitive impairment in a real clinical setting. Fifteen patients with amnestic mild cognitive impairment were enrolled in this prospective observational study in three Korean medicine hospitals. Korean medicine treatments were delivered by experienced professionals and not restricted to standardized treatment. Outcome measures were prospectively planned to examine the Korean-Montreal Cognitive Assessment (K-MoCA), Korean-Mini Mental State Examination (K-MMSE), and other detailed neuropsychological assessment at the baseline and after 12 and 24 weeks of treatment. Korean medicine treatment for MCI treatment in the real-world clinical setting included herbal medicine and acupuncture. The most frequently used herbs in herbal decoctions were *Acori Graminei Rhizoma*, *Polygalae Radix*, and *Poria Sclerotium Cum Pini Radix*. The herbal medicine formulae used in this study were classified into three categories: tonifying Qi (33.3%), tonifying kidney (46.7%), and calming liver (20%) formulae. In the cognitive ability assessment, the K-MoCA score significantly improved after treatment (mean difference 2.6; 95% CI: 1.3 to 3.9, *p*=0.001). The K-MMSE score slightly increased after treatment; however, the improvement was not statistically significant (mean difference 0.8; 95% CI: −0.5 to 2.0, *p*=0.195). In detailed neuropsychological assessment, the cognitive domains of executive functions and memory after the treatment were distinctively improved. In this prospective observational case series, we could see the real clinical environments of treating patients with mild cognitive impairment in Korean medicine hospitals. Patients treated with Korean medicine showed improved results in the neuropsychological assessment after 12 and 24 weeks.

## 1. Introduction

Mild cognitive impairment (MCI) is a transitional state between normal aging and early dementia. The diagnostic and statistical manual of mental disorders-5 (DSM-5) distinguishes mild neurocognitive disorder from major neurocognitive disorder. Mild neurocognitive disorder is defined by a noticeable decrement in cognitive functioning that goes beyond normal changes seen in aging but that does not interfere with the independence of the individual in relation to everyday activities [1–3]. MCI is clinically important, in that it elevates the risk of progression for dementia. In the elderly aged 65 or older, 46% of people with mild cognitive impairment develop dementia, while 3% of normal people develop dementia within 3 years [4]. Early detection of mild neurocognitive impairment and prevention of its progression may ease the burden of major neurocognitive disorder.

There is no recommended conventional treatment for MCI with sufficient evidence, and representatively, cholinesterase inhibitors did not show consistent effectiveness for MCI patients in the systematic review [1]. The need for effective therapies treating MCI is increasing, and the potential effectiveness of acupuncture and herbal medicine for MCI is receiving attention. Numerous studies have reported that acupuncture is effective in treating MCI and can be an alternative and adjunctive treatment for MCI patients [5–7]. There have been many randomized controlled trials [8–11] and systematic reviews [12–14] of Chinese herbal medicine for MCI treatment. Although the randomized controlled trial and systematic review are good strategies to provide high quality evidence with low risk of bias, they have some restrictions in reflecting the current healthcare environments. Studies exploring which treatment is applied in the real clinical setting, and which domain is improved after treatment, are also required for the purpose of reflecting reality.

In the South Korean healthcare system, patients with mild cognitive impairment can choose to receive the medical care of Korean traditional medicine for the prevention and management of cognitive disorders in the dual medical system of Western medicine and Korean medicine (KM) [15]. Most KM hospitals run dementia clinics by certified neuropsychiatrists of KM [16]. The integrative treatments used in the dementia clinic in KM hospitals include acupuncture, herbal medicine, and cupping therapy. Some public health centers in South Korea have an MCI management program using herbal medicines that showed successfully improved MCI symptoms [17].

To observe the variation in treatment options and patient-specific treatments, observational studies or pragmatic trials with no restriction on the treatment are needed. For this purpose, we designed a prospective, observational, case series to explore the effectiveness and real-world usage of KM for MCI patients.

## 2. Materials and Methods

### 2.1. Study Design

This study is a multicenter prospective observational case series conducted by three university KM hospitals. The names of the three hospitals are labelled in this report as A, B, and C hospitals, respectively. This study was conducted in accordance with the study protocol approved by the institutional review boards of Kyung Hee University Korean Medicine Hospital (KOMCIRB-150901-HR-036), Wonkwang University Sanbon Hospital (WMCSB 2016-51-1635), and Dunsan Korean Medicine Hospital of Daejeon University (DJDSKH-16-BM-13). Written informed consent was obtained from all participants before the procedures. The protocol was retrospectively registered with the clinical research information service (KCT0002322).

### 2.2. Participants

Eligible patients were recruited in three KM university hospitals in the outpatient setting from December 07, 2016, to March 29, 2017. The inclusion criteria included that patients met the Petersen diagnostic criteria of MCI [18], were aged from equal to or more than 45 years of age, and agreed to participate with written informed consent. The following patients were excluded: (1) history of cognitive impairment due to any other causes (for example, head trauma or brain injury); (2) brain disorders including Parkinson's disease, Huntington's Disease, normal pressure hydrocephalus, or brain tumor; (3) cardiovascular disease, endocrinopathy, or gastrointestinal tract disorders not controlled by diet therapy and drug treatment; (4) diabetic not controlled by hypoglycemic agents or insulin; (5) seriously unstable medical condition; (6) severe kidney disease or liver disease; (7) anemia, hypothyroidism, vitamin deficiencies, or malignancy; (8) any history of drug or alcohol dependence during the past 6 months; (9) history of major psychiatric disorders, such as schizophrenia, delusional disorder, depression, bipolar disorder alcohol, or substance abuse disorders; (10) involved in other clinical trials within 4 weeks; (11) pregnant, breastfeeding, or inadequate contraception; (12) mental retardation, emotional, or intellectual problems and difficulty in understanding the research; (13) blindness, hypacusis, or dysphonia; (14) not eligible for the clinical research in accordance with the researcher's judgement.

### 2.3. Procedures

All patients received KM treatment by experienced professionals in three university hospitals. Because the aim of the study was to observe the real clinical setting in a KM hospital, interventions were not restricted to a fixed protocol, and the clinicians in the three university hospitals were free to choose treatment options for patients with MCI. The applied treatment details, including the composition of the prescribed herbal formula and the acupuncture points, were recorded in detail at every visit. To determine the effectiveness of the KM treatment, an evaluation method was prospectively planned. Patients received fixed neuropsychological assessment battery at the baseline and at the 12^th^ and 24^th^ week of treatment.

### 2.4. Outcome Measurement

The Korean-Montreal Cognitive Assessment (K-MoCA) [19, 20] and Korean-Mini Mental State Examination (K-MMSE) [21, 22] were examined at the baseline and after the 12 and 24 weeks of treatment. For the detailed neuropsychological assessment, Seoul neuropsychological screening battery (SNSB) was also done on the same day. SNSB is composed of five domains: attention, language and related functions, visuospatial functions, memory, and frontal/executive functions [23, 24]. Digit span test (DST) for the attention domain [25], Korean-Boston Naming Test (K-BNT) for the language domain [26], Rey Complex Figure Test (RCFT) [27], and Seoul Verbal Learning Test (SVLT) [28] for the visuospatial domain and the memory domain, respectively, Contrasting Program and Go-No-Go Test for the frontal function, and Korean-Color Word Stroop Test (K-CWST) [29] and Controlled Oral Word Association Test (K-COWAT) [30] for the executive function domain are included in the battery.

### 2.5. Safety Assessment

For safety assessment, every adverse event was carefully documented during the study, and the laboratory parameters related to liver function (aspartate aminotransferase, alanine aminotransferase, and total bilirubin), kidney function (blood urea nitrogen and creatinine), and thyroid function (thyroid stimulating hormone and free thyroxine) were analyzed by the blood test at the baseline and at the 12^th^ and 24^th^ week of treatment.

### 2.6. Statistical Analysis

Data are shown in mean ± standard deviation. Depending on the normality of the data, a paired *t*-test or Wilcoxon signed rank test was used to evaluate the efficacy of the KM treatment for patients with MCI. The Shapiro–Wilk normality test was done to test the normality of the data. A *p*-value of less than 0.05 was considered to represent statistical significance. The software used for all the statistical analyses was R version 3.6.0. Network analysis and visualization of the frequently used herbs in decoctions were also done using the software R version 3.6.0. Association rule mining was used to score the support between herbs with the *arules*, the R extension package [31]. Then, network visualization was done with the *igraph*, another extension package of R, with nodes for each herb and edges for the associations between herbs. We also conducted community detection, which can distinguish groups according to the density of connection, by the Newman–Girvan algorithm [32, 33].

## 3. Results

### 3.1. Baseline Characteristics

**Figure 1:**
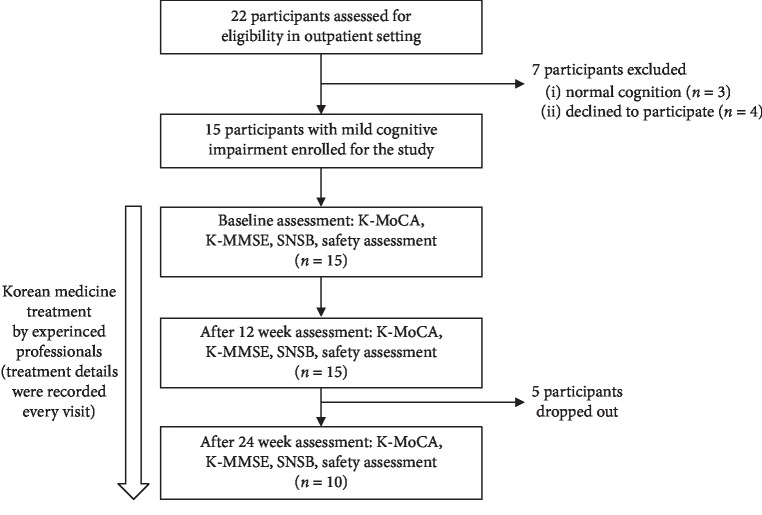
Participant flow chart. Participants received Korean medicine treatment by experienced professionals from the baseline to after 24 weeks. Korean medicine treatment was not restricted to standardized treatment, and the outcome assessments were prospectively planed.

Twenty-two participants were screened for eligibility, and fifteen patients diagnosed with MCI were included from the three KM universities. The patients visited hospital every two weeks, to be prescribed herbal medicine. Some patients had additional visits for acupuncture treatment. Among the total of 15 patients, 80% were women and 20% were men. The mean age and education level were 64.5 ± 10.0 and 8.3 ± 3.2 years, respectively. The type of MCI was amnestic MCI in all participants based on Peterson's criteria [34] of having a memory complaint and objective memory impairment for the patient's age. The average K-MoCA score was 19.7 ± 3.6, and the K-MMSE score was 25.9 ± 2.4. In a previous study of MCI patients conducted in Korea [35], the average K-MoCA score was 18.5 ± 3.7 and the average K-MMSE score was 24.0 ± 2.9, which were similar to the results of our study. The demographical distribution and baseline neuropsychological assessment score of patients were similar among the three hospitals (Table 1). Five patients terminated their treatment between 12 and 24 weeks of treatment for personal reasons.  Figure 1 shows the flow chart outlining the study design.

### 3.2. Herbal Medicine Treatments Used for MCI Patients

**Figure 2:**
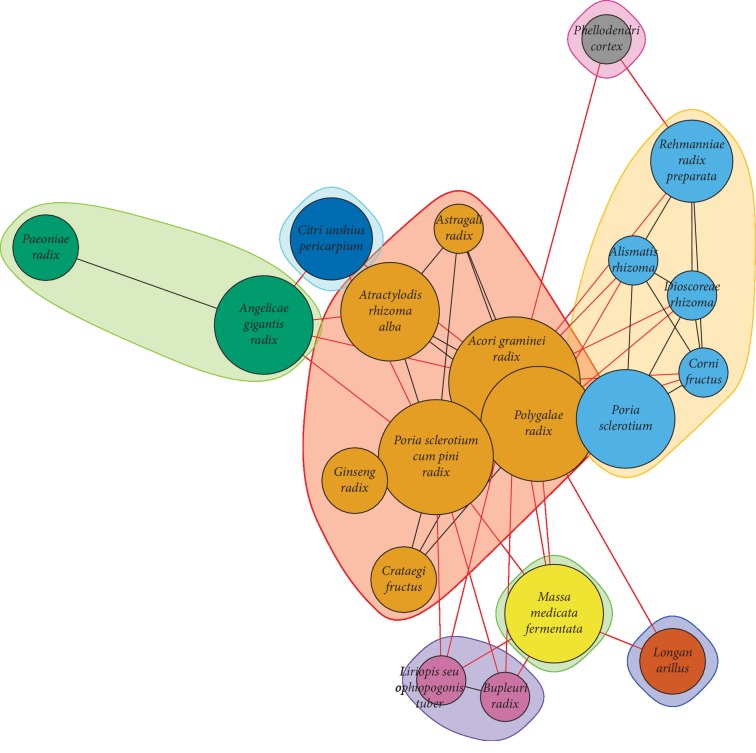
Network visualization of frequently used herbs in the herbal decoction for patients with mild cognitive impairment. The size of the nodes represents the frequency of herb used in the decoction, while the distance between nodes represents the association between herbs. Grouping was done with community detection, and as a result, the large orange nodes in the core are composed of herbs frequently used for MCI, regardless of the KM type. The blue nodes were mainly composed of tonifying-kidney herbs, whereas the green and purple nodes seem to be calming-liver herbs.

Table 2 summarizes the KM treatments applied for patients with MCI in this study, and [Supplementary-material supplementary-material-1] of the Supplementary Information (SI) provides a detailed description of each treatment option provided. All formulae were used in the modified form, and specific herbs for memory impairment were added to the standard formula. The commonly added herbs are presented in Table 2 below the frequency of the formula used.  Figure 2 presents network visualization of the frequently used herbs in the herbal decoction formulae and their grouping by community detection. The frequency of each herb in decoction is represented by the size of node, and the association between herbs is represented by the distance of nodes.

Table 2 shows that the herbs most frequently added to the standard herbal decoction were Acori Graminei Rhizoma (石菖蒲), followed by Poria Sclerotium Cum Pini Radix (茯神), Polygalae Radix (遠志), and Massa Medicata Fermentata (神麯), regardless of the KM pattern. As for the standard formula used, of the 15 total patients, 5 patients were prescribed herbal medicine with tonifying Qi formulae, 7 patients were prescribed herbal medicine with tonifying kidney formulae, and 3 patients were prescribed herbal medicine with calming liver formulae.

As shown in Figure 2, Acori Graminei Rhizoma (石菖蒲), Poria Sclerotium Cum Pini Radix (茯神), and Polygalae Radix (遠志) are located in the core part (large orange nodes in the center) of the network, which were the herbs most frequently added to the formulae of the patients with MCI.

### 3.3. Acupuncture and Other Treatments Used for MCI Patients

Acupuncture treatments in A hospital and B hospital were initially planned to be twice a week, but the treatment frequency gradually decreased by time, depending on the compliance of each patient. Acupuncture points on the head, hands, and feet were used, and for some patients, electric stimulation was added. GV20 (百會) in the head was the most commonly used acupuncture point, and the other points on the hands and feet varied, depending on the clinicians. Besides acupuncture and herbal medicine, other treatment methods, such as pharmacopuncture, cupping therapy, aroma therapy, and moxibustion, were added. Pharmacopuncture is a form of acupuncture technique that injects herbal extract into the acupuncture point. Hominis placental pharmacopuncture was additionally injected at the lower abdomen after acupuncture treatment to improve memory impairment. Three patients were additionally given aroma therapy during acupuncture treatment to improve the effects of the acupuncture. Another three patients received cupping therapy irregularly, mainly on the upper back region to alleviate pain and tightness in the applied region. There was also one patient who received moxibustion treatment to the abdominal region, after or before acupuncture treatment, to improve digestive function.

### 3.4. Cognitive Function: K-MoCA and K-MMSE

The total K-MoCA score was 19.73 ± 3.59 at the baseline, and after 12 and 24 weeks, the total K-MoCA score was increased to 22.33 ± 5.09 and 23.60 ± 6.04, respectively. These differences were statistically significant (*p*=0.015 and *p*=0.008, respectively) (Table 3 and [Supplementary-material supplementary-material-1] in the Supplementary Materials). For the seven subcategory scores, overall scores tended to increase over time, and the improvement of the visuospatial/executive function was distinct. The total K-MMSE score at the baseline and after the 12 and 24 weeks of treatment was, respectively, 25.87 ± 2.39, 26.67 ± 3.15, and 26.20 ± 4.02. These differences were not statistically significant with a *p* value of 0.195 and 0.761, respectively (Table 3 and [Supplementary-material supplementary-material-1] in the Supplementary Materials).

### 3.5. Neuropsychological Assessment: Seoul Neuropsychological Screening Battery (SNSB)

In the digit span test, the score tended to increase slightly throughout the study, but the difference was statistically significant only in the digit span test—backward on the 12^th^ week, compared to the baseline (*p*=0.013). K-BNT score was 46.60 ± 7.58 at the baseline, and it improved to 47.53 ± 8.41 and 50.70 ± 9.33 after the 12 and 24 weeks, respectively. The difference between the baseline and after 24 weeks of treatment was statistically significant (*p*=0.013). For the visuospatial function, the RCFT copy score did not change (*p*=0.823 and *p*=0.335, respectively), but the copy time after 24 weeks was significantly decreased from 160.07 ± 51.55 to 130.00 ± 52.07 (*p*=0.050).

For the memory test, the immediate recall and delayed recall score showed the tendency of improving gradually after the treatment in both verbal and visual memory. However, recognition scores were not improved in either the verbal or the visual memory test. For the motor regulation and perseveration, contrasting program and Go-No-Go test scores were high at the baseline and did not get worse throughout the periods. In K-COWAT, the scores increased significantly after 12 weeks in both semantic and phonemic word fluency (*p*=0.012 and *p*=0.003), but the increases were not significant after 24 weeks (*p*=0.104 and *p*=0.232). In K-CWST, the numbers of correct response in word reading tended to increase but were not statistically significant (*p*=0.422 and *p*=0.208). However, the number of correct responses in color reading after the treatment was improved (*p*=0.016 and *p*=0.014) (Table 4).

### 3.6. Safety Assessment

Two patients reported mild indigestion and were recovered without any additional treatment. No serious adverse event was observed during the study periods. Also, there was no clinically significant change in the blood test. Levels of alanine aminotransferase (ALT) showed a tendency to decrease during the treatment periods, but the changes were not statistically significant (Table 5).

## 4. Discussion

In this prospective observational case series, we analyzed the real-world use of Korean medicine therapy and its outcomes in the treatment of patients with mild cognitive impairment. Our results showed that cognitive ability assessments, such as K-MoCA and K-MMSE scores, improved after KM treatment. Interestingly, the herbal prescriptions were based on KM patterns, but the most frequently used herbs were used to target symptoms, such as cognitive impairment, regardless of the KM pattern. The most frequently used herbs for patients with MCI were *Acori Graminei Rhizoma*, *Polygalae Radix*, and *Poria Sclerotium Cum Pini Radix*, regardless of the KM patterns. Also, they were the most frequently added herbs to the basic formulae (water extract) for treating patients with MCI. These three herbs are components of the three-herb formula named Smart Soup (Chongmyung-tang in Korean and Congming-tang in Chinese), which has been traditionally applied for aging-related cognitive impairment for many centuries and showed antiamnesic effects in various mice models of memory impairment [36, 37]. In a previous retrospective cohort study conducted in Ruijin Hospital in China, combination therapy with this herbal formula and Aricept showed a better therapeutic outcome than monotherapy with Aricept only in patients with Alzheimer's disease [38]. These three herbs correspond exactly with those in a previous review of Chinese herbs for memory disorders based on the classical herbal literature [39]. When clinicians choose herbs for patients with MCI, these three herbs can be recommended based on previous research and clinical experience.

The list of frequently used herbs was similar to a previous study that reported a specific list of herbs for age-related dementia based on traditional Chinese literature [40]. The 10 most frequently used herbs in the formulae for age-related memory disorders found in the previous study were all included in our list of the top 20 frequently used herbs. Another previous study identified five herbal species for memory impairment based on the *Encyclopedia of Traditional Chinese Medicine* database, including *Poria cocos*, *Polygala tenuifolia*, *Rehmannia glutinosa*, *Panax ginseng*, and *Acorus* species, which were all included in our list [41]. Also, a dendrogram of hierarchical clustering of herbs in a previous study found three clusters of herbs. The first cluster was composed of the four herbs, of *Acori Graminei Rhizoma*, *Polygalae Radix*, *Poria Sclerotium Cum Pini Radix*, and *Ginseng Radix* [40]. These four herbs are also located in the core part in the network visualization figure using the data from real clinical cases. Only the location of *Poria Sclerotium* (茯笭, *Fu ling*) and *Poria Sclertium Cum Pini Radix* (茯神, Fu shen) was different between the two cluster analysis results. Furthermore, these four herbs are ingredients in the Kai xin san formula, which has been recommended for preventing memory loss in old age [41] and reported to have a potential of neuroprotective effect and improved cognitive function [42, 43].

Among the individual herbs for memory impairment, *Acori Graminei Rhizoma* is known to have a protective effect on cognitive impairment in animal studies [44, 45]. In a randomized controlled trial comparing Chinese medicine and placebo for amnestic MCI patients, *Polygalae Radix* was the representative herb for tonifying the kidney and resolving phlegm and blood stasis formula for treating MCI in a randomized controlled trial [8]. A study using Alzheimer's disease transgenic mice showed an ameliorating effect for cognitive impairment of *Poria Sclerotium Cum Pini Radix*-containing formula [37]. However, the other herbs mentioned above were used to improve cognitive function, and *Massa Medicata Fermentata* was used to help digestive function. Experienced KM professionals noted that this herb was selected to enhance the medicinal effect of herbal medicine by helping digestion. Little evidence regarding the use of *Massa Medicata Fermentata* for cognitive disorders has been presented. In a review paper on herbal medicine for dementia, *Massa Medicata Fermentata-*containing formulae were used five times in experimental studies of dementia [46]. In addition, *Massa Medicata Fermentata-*containing formulae were reported to have intestinal protective effects in rats [47]. Clinicians may consider the use of *Massa Medicata Fermentata* to enhance the effect of other herbal medicines; however, further research is needed.

Herbal medicine formulae used for treating MCI were under the categories of three KM pattern types: tonifying Qi, tonifying kidney, and calming liver. Previous study of Chinese medicine syndrome among vascular MCI reported that there are eight types of pattern that comprise Qi deficiency, Qi stagnation, blood deficiency, blood stasis, phlegm-dampness, fire-heat, Yang deficiency, and Yin deficiency [48]. Other studies suggested that the kidney essence deficiency, phlegm stasis, and blood stasis are the main pathophysiological mechanisms of MCI in traditional Chinese medicine [8]. In the KM Pattern Identification for Dementia, MCI patients can be classified into four KM pattern types, which are Qi deficiency, Yin deficiency, dampness-phlegm, and fire-heat patterns [49]. Taken together, Qi deficiency and Yin (kidney) deficiency are the most common TCM pattern types in patients with cognitive impairment. This is in line with our study, in the sense that 80% of MCI patients were prescribed with Qi deficiency or kidney deficiency formulae.

There were some variations in treatment option depending on the practitioners. In A hospital, patients received herbal decoction (water extract) for the first 6 weeks, and then herbal medicine changed to manufactured granules for the remaining weeks. On the contrary, all patients in C hospital were prescribed manufactured granules during whole periods. Most patients in A and B hospitals were planned to receive acupuncture treatment, while patients in C hospital only received herbal medicine treatment for managing mild cognitive disorder.

Due to the small sample size of this observational case series, there were some limitations for comparison between each treatment method. However, we attained the lists of frequently prescribed herbal medicine according to the KM patterns. In tonifying Qi formulae, Bojungikgi-tang (Buzhongyiqi-tang in Chinese and Hochuekki-To in Japanese) was the most representative formula, and *Ginseng Radix* was the common herb. Bojungikgi-tang showed antidementia and neuroprotective effect in Aβ-injected mice [50] and improved the symptoms of vascular dementia in some case reports [51, 52]. Also, Guibi-tang (Guipi-tang in Chinese and Kihi-to in Japanese) showed the effect on memory deficit induced by Aβ [53] and improved cognitive function in patients with Alzheimer's disease [54] in previous studies. In tonifying kidney formulae, a number of studies of tonifying kidney (Bushen in Chinese) formula for cognitive impairment have been reported, mainly concentrated in China [8, 10, 11, 55]. Bushen capsule, a representative Chinese medicine formula for cognitive disorder, includes *Cistanchis Herba* and *Alismatis Rhizoma* [10, 11], and in our study, *Alismatis Rhizoma* was used in some tonifying kidney decoction and manufactured granule. In calming liver formulae, herbal decoctions, which mainly include *Cyperi Rhizoma*, *Bupleuri Radix*, and *Uncariae Ramulus cum Uncus*, were also used in this study. Ukgan-san (Yigan-san in Chinese and Yokukan-san in Japanese), containing *Bupleuri Radix* and *Uncariae Ramulus cum Uncus*, is a typically used formula in treatment for the behavioral and psychological symptoms of dementia (BPSD) [56, 57]. In addition, another calming-liver formula, Dangguijagyag-san is reported in a previous observational study to improve cognitive ability in patients diagnosed with MCI [58].

Although herbal decoctions and granules were the mainly used treatment for MCI patients in KM hospital, acupuncture and other treatments, including pharmacopuncture, cupping therapy, moxibustion, and aroma therapy, were being used in clinic as well. GV20 was a commonly used acupuncture point in acupuncture treatment, which is consistent with those of previous studies [7].

After the KM treatment, the cognitive ability of patients measured by K-MoCA improved. However, the K-MMSE score did not show statistically significant improvement. This may be due to the ceiling effect. Because the score of K-MMSE in our study was relatively high from the beginning, there was a limitation to reflecting the treatment effect. For this ceiling effect, K-MoCA is reported to be a better detector of early cognitive dysfunction [59]. According to a systematic review of Chinese herbal medicine for MCI, the MMSE score showed inconsistent results. The MMSE score was not significantly improved compared to placebo in studies with 12 weeks of treatment period, whereas it significantly improved in studies with 16 weeks of treatment period [13]. By contrast, systematic review with MoCA score showed more consistent result in the effect of herbal medicine for MCI. In another systematic review, Chinese herbal medicine improved the MoCA score by 1.76–2.34 points compared to placebo after 24 weeks of treatment, which was statistically significant [12].

There have been few studies observing detailed neuropsychological assessment after herbal medicine or acupuncture treatment, which may be due to time and cost constraints. The strengths of this study are that our results showed noticeable improvements in the specific cognitive domains after KM treatment and that we used detailed neuropsychological assessment methods for this observational case series. When planning the treatment strategy for MCI patients, it is important to know which treatment is effective for the management of each domain or part in cognitive ability. The current study showed that the KM treatment was particularly effective in the improvement of executive functions and delayed recall, both in score of K-MoCA and SNSB. As there were no serious adverse events or change in the blood test, KM treatment seems to be safe based on our limited cases.

There are some limitations to our study. First, it is an observational case series without a control group, and the effect size of KM treatment for MCI in our study has the potential to be overestimated. Secondly, due to the small sample size, it was not sufficient to compare the difference in the effects among tonifying Qi, tonifying kidney, or calming liver formula. In the future, pragmatic trial of KM treatment for MCI with a large sample size is needed. Third, practitioners were free to choose treatment option and herbal formula in our study, but our study did not collect data about the detailed process of pattern identification and reasons for the prescription. Further studies are required to evaluate the effectiveness of each personalized herbal treatment based on KM patterns.

## 5. Conclusions

In the present observational case series of KM treatment for patients with MCI, we could observe the patient-specific treatments and detailed compositions of each treatment that have been applied in the KM hospital by experienced professionals without any restrictions. Also, we could explore the specific domain in cognitive ability improved by the KM treatment in a real clinical setting. The results from this prospective, observational, case series may provide useful information for further researchers and clinicians for determining treatment options.

## Figures and Tables

**Table 1:** Baseline characteristics of patients with mild cognitive impairment (*n* = 15).

	A hospital (*n* = 8)	B hospital (*n* = 2)	C hospital (*n* = 5)	Total (*n* = 15)	*p* value
Age	65.6 ± 6.3	49.0 ± 0.0	68.8 ± 11.5	64.5 ± 10.0	0.738
Female, % (*n*)	87.5 (7)	50 (1)	80 (4)	80.0 (12)	0.495
Education level	8.5 ± 1.9	10.5 ± 1.4	7.2 ± 3.4	8.3 ± 3.2	0.324
K-MoCA	18.6 ± 2.3	23.5 ± 0.7	20.0 ± 5.1	19.7 ± 3.6	0.441
K-MMSE	24.9 ± 2.3	28.0 ± 1.4	26.6 ± 2.3	25.9 ± 2.4	0.176
SGDS	6.4 ± 4.3	4.0 ± 0.0	4.2 ± 3.3	5.3 ± 3.7	0.296
B-ADL	19.9 ± 0.4	20 ± 0.0	19.8 ± 0.4	19.9 ± 0.4	0.777
K-IADL	2.2 ± 1.5	2.0 ± 1.4	2.4 ± 3.7	2.3 ± 2.3	0.925

Values are expressed as mean ± standard deviation. K-MoCA, Korean-Montreal Cognitive Assessment; K-MMSE, Korean-Mini Mental State Examination; SGDS, Short version of the Geriatric Depression Scale; B-ADL, Barthel-Activities of Daily Living; K-IADL, Korean-Instrumental Activities of Daily Living.

**Table 2:** Korean medicine treatment used for patients with mild cognitive impairment.

	No. of participants (%)
Herbal medicine	15 (100.00)
Decoction (water extract)^a^	9 (60.00)
**Tonifying Qi formula**	**2 (13.33)**
Modified Jeungsonbaekchul-san and Gakbyeongyeonsu-tang	1 (6.67)
Modified Guibi-tang	1 (6.67)
**Tonifying kidney formula**	**4 (26.67)**
Modified Jaeumganghwa-tang	2 (13.33)
Modified Yukmijihwang-hwan	1 (6.67)
Modified Gojinenmja	1 (6.67)
**Calming liver formula**	**3 (20.00)**
Modified Ondam-tang	1 (6.67)
Modified Soyo-san	1 (6.67)
Modified Daeyoung-jeon	1 (6.67)
**Herbs added to the standard formula**^b^	
*Acori Graminei Rhizoma*	7 (46.67)
*Poria Sclertium Cum Pini Radix*	6 (40.00)
*Polygalae Radix*	6 (40.00)
*Massa Medicata Fermentata*	6 (40.00)
Granules (manufactured)	14 (93.33)
**Tonifying Qi formula**	**5 (33.33)**
Bojungikgi-tang	5 (33.33)
**Tonifying kidney formula**	**7 (46.67)**
Uchashinki-hwan	7 (46.67)
**Calming liver formula**	**2 (13.33)**
Ukgan-san	2 (13.33)

Acupuncture	7 (46.67)
Electroacupuncture	1 (6.67)
Pharmacopuncture	1 (6.67)
Cupping	3 (20.00)
Moxibustion	1 (6.67)
Aroma therapy	3 (20.00)

^a^All formulae were used in the modified form, and detailed descriptions of the herbs used in each formula are provided in [Supplementary-material supplementary-material-1] of the Supplementary Information. Herbs frequently added to the standard formula are presented in Table 2 below the frequency of the formula used. The Chinese and Japanese names of each formula are also presented in [Supplementary-material supplementary-material-1]. ^b^Herbs added to the standard formula more than six times are shown in this table. The other herbs were added to the standard formula less than six times.

**Table 3:** Changes in the Korean-Montreal Cognitive Assessment (K-MoCA) and Korean-Mini Mental State Examination (K-MMSE).

	Baseline (*n* = 15)	After 12 weeks (*n* = 15)	After 24 weeks (*n* = 10)	*p* value	
				12 wk^a^	24 wk^b^
K-MoCA					
Visuospatial/executive function	3.13 ± 1.19	3.80 ± 1.26	4.50 ± 0.97	0.015	0.008
Naming	2.73 ± 0.59	2.53 ± 0.74	2.70 ± 0.48	0.149	0.773
Attention	3.93 ± 1.44	4.67 ± 1.29	4.80 ± 1.62	0.036	0.591
Language	2.27 ± 0.70	2.60 ± 0.51	2.60 ± 0.70	0.037	0.424
Abstraction	1.27 ± 0.59	1.53 ± 0.52	1.44 ± 0.73	0.129	1.000
Delayed recall	1.07 ± 1.28	1.87 ± 1.25	2.10 ± 1.97	0.068	0.026
Orientation	5.13 ± 0.99	5.13 ± 1.55	5.30 ± 1.49	1.000	0.443
Total score	19.73 ± 3.59	22.33 ± 5.09	23.60 ± 6.04	0.001	0.021

K-MMSE					
Orientation to time	4.27 ± 1.10	4.47 ± 1.13	4.30 ± 1.49	0.233	0.424
Orientation to place	4.80 ± 0.41	4.73 ± 0.80	4.70 ± 0.67	1.000	0.850
Registration	3.00 ± 0.00	3.00 ± 0.00	3.00 ± 0.00	NA	NA
Attention and calculation	3.80 ± 1.15	4.27 ± 1.03	3.80 ± 1.23	0.131	1.000
Recall	1.13 ± 0.83	1.20 ± 0.94	1.40 ± 0.97	0.777	0.265
Language	7.67 ± 0.62	7.87 ± 0.35	7.90 ± 0.32	0.374	1.000
Drawing	1.00 ± 0.00	0.93 ± 0.26	0.80 ± 0.42	1.000	0.346
Total score	25.87 ± 2.39	26.60 ± 3.01	26.20 ± 4.02	0.215	0.761

Values are expressed as mean ± standard deviation. *p* values are calculated by Student's paired *t*-test or Wilcoxon signed rank test, depending on the normality. ^a^Comparing measurement before the intervention (baseline) and after 12 weeks. ^b^Comparing measurement before the intervention (baseline) and after 24 weeks. K-MoCA, Korean-Montreal Cognitive Assessment; K-MMSE, Korean-Mini Mental State Examination.

**Table 4:** Changes in detailed neuropsychological assessment.

	Baseline (*n* = 15)	After 12 weeks (*n* = 15)	After 24 weeks (*n* = 10)	*p* value	
				12 wk^a^	24 wk^b^
Attention					
Digit span test: forward	5.53 ± 1.46	5.87 ± 1.36	6.30 ± 1.77	0.142	0.120
Digit span test: backward	3.20 ± 1.21	4.07 ± 1.53	3.80 ± 1.62	0.013	0.089

Language					
K-BNT	46.60 ± 7.58	47.53 ± 8.41	50.70 ± 9.33	0.347	0.013

Visuospatial functions					
RCFT copy score	28.83 ± 8.31	28.53 ± 8.42	29.20 ± 10.24	0.823	0.335
RCFT copy time	160.07 ± 51.55	161.60 ± 85.77	130.00 ± 52.07	0.921	0.050

Memory					
Verbal memory					
SVLT immediate recall	16.13 ± 4.50	18.40 ± 6.68	20.40 ± 6.88	0.026	0.022
SVLT delayed recall	4.20 ± 2.70	4.93 ± 3.56	5.60 ± 3.37	0.208	0.025
SVLT recognition	16.13 ± 4.41	16.93 ± 5.08	15.50 ± 6.93	0.351	0.754
Visual memory					
RCFT immediate recall	9.97 ± 7.46	12.23 ± 9.15	17.50 ± 10.68	0.088	0.018
RCFT delayed recall	9.17 ± 6.50	12.63 ± 8.91	17.25 ± 10.77	0.005	0.030
RCFT recognition	14.93 ± 5.18	16.60 ± 5.45	15.20 ± 7.45	0.092	0.123

Frontal/executive functions					
Contrasting program	18.67 ± 2.87	18.67 ± 3.13	18.80 ± 3.46	1.000	0.174
Go-No-Go	17.87 ± 2.67	18.47 ± 2.75	17.30 ± 5.25	0.324	1.000
K-COWAT					
Semantics fluency	28.67 ± 8.31	32.80 ± 8.90	32.50 ± 12.78	0.012	0.104
Phonemics fluency	19.20 ± 10.12	21.87 ± 10.82	22.80 ± 14.27	0.003	0.232
K-CWST					
Word reading: correct	105.60 ± 10.53	107.67 ± 9.00	109.10 ± 8.48	0.422	0.208
Word reading: time per item	0.86 ± 0.32	0.80 ± 0.25	0.80 ± 0.25	0.318	0.156
Color reading: correct	68.60 ± 29.82	79.93 ± 26.57	83.90 ± 31.16	0.016	0.014
Color reading: time per item	1.85 ± 0.83	1.56 ± 0.57	1.45 ± 0.60	0.132	0.083

Values are expressed as mean ± standard deviation. *p* values are calculated by Student's paired *t*-test or Wilcoxon signed rank test, depending on the normality. ^a^Comparing measurement before the intervention (baseline) and after 12 weeks. ^b^Comparing measurement before the intervention (baseline) and after 24 weeks. K-BNT, Korean-Boston Naming Test; RCFT, Rey Complex Figure Test; SVLT, Seoul Verbal Learning Test; K-COWAT, Korean version of Controlled Oral Word Association Test; K-CWST, Korean version of Color Word Stroop Test.

**Table 5:** Changes in laboratory parameters by the blood test.

	Baseline (*n* = 15)	After 12 weeks (*n* = 15)	After 24 weeks (*n* = 10)	*p* value	
				12 wk^a^	24 wk^b^
AST (mg/dL)	27.40 ± 11.69	26.73 ± 11.55	26.50 ± 7.01	0.865	0.672
ALT (mg/dL)	30.40 ± 20.10	25.20 ± 20.58	22.70 ± 15.15	0.362	0.053
Total bilirubin (mg/dL)	0.58 ± 0.19	1.00 ± 1.52	0.54 ± 0.14	0.307	0.192
Albumin (g/gL)	4.24 ± 0.17	4.20 ± 0.20	4.25 ± 0.36	0.452	0.906
BUN (mg/dL)	17.33 ± 5.96	17.46 ± 4.71	19.42 ± 6.66	0.902	0.575
Creatinine (mg/dL)	0.77 ± 0.20	0.79 ± 0.19	0.84 ± 0.30	0.239	0.086
TSH (mIU/L)	2.00 ± 1.34	2.35 ± 1.05	1.77 ± 0.86	0.233	0.185
Free T4 (ng/dL)	1.19 ± 0.22	1.15 ± 0.16	1.24 ± 0.22	0.382	0.236
ESR (mm/hr)	12.27 ± 7.33	12.00 ± 6.45	10.00 ± 8.67	0.871	0.622

Values are expressed as mean ± standard deviation. *p* values calculated by Student's paired *t*-test or Wilcoxon signed rank test depending on normality. ^a^Comparing measurement before the intervention (baseline) and after 12 weeks. ^b^Comparing measurement before the intervention (baseline) and after 24 weeks. AST, aspartate aminotransferase; ALT, alanine aminotransferase; BUN, blood urea nitrogen; TSH, thyroid stimulating hormone; Free T4, free thyroxine; ESR, erythrocyte sedimentation rate.

## Data Availability

The data used to support the findings of this study are available from the corresponding author upon request.

## Abbreviations

DST: Digit span test
K-BNT: Korean-Boston Naming Test
K-COWAT: Controlled Oral Word Association Test
K-CWST: Korean-Color Word Stroop Test
KM: Korean medicine
K-MMSE: Korean-Mini Mental State Examination
K-MoCA: Korean-Montreal Cognitive Assessment
MCI: Mild cognitive impairment
RCFT: Rey Complex Figure Test
SNSB: Seoul Neuropsychological Screening Battery
SVLT: Seoul Verbal Learning Test.

## Supplementary Materials

Supplementary Materials Supplementary Figure S1: changes in total scores of the Korean-Montreal Cognitive Assessment (K-MoCA) and the Korean-Mini Mental State Examination (K-MMSE). Supplementary Table S1: detailed descriptions of the Korean medicine treatment used in this study.
