# Supplementary material for: Korean Traditional Medicine in Treating Patients with Mild Cognitive Impairment: A Multicenter Prospective Observational Case Series
Source: Evid Based Complement Alternat Med. 2020 Feb 22;2020:4323989. doi: 10.1155/2020/4323989 (PMC7060453; doi:10.1155/2020/4323989)
Supplement: Supplementary Materials — Supplementary Figure S1: changes in total scores of the Korean-Montreal Cognitive Assessment (K-MoCA) and the Korean-Mini Mental State Examination (K-MMSE). Supplementary Table S1: detailed descriptions of the Korean medicine treatment used in this study. [file 4323989.f1.pdf]

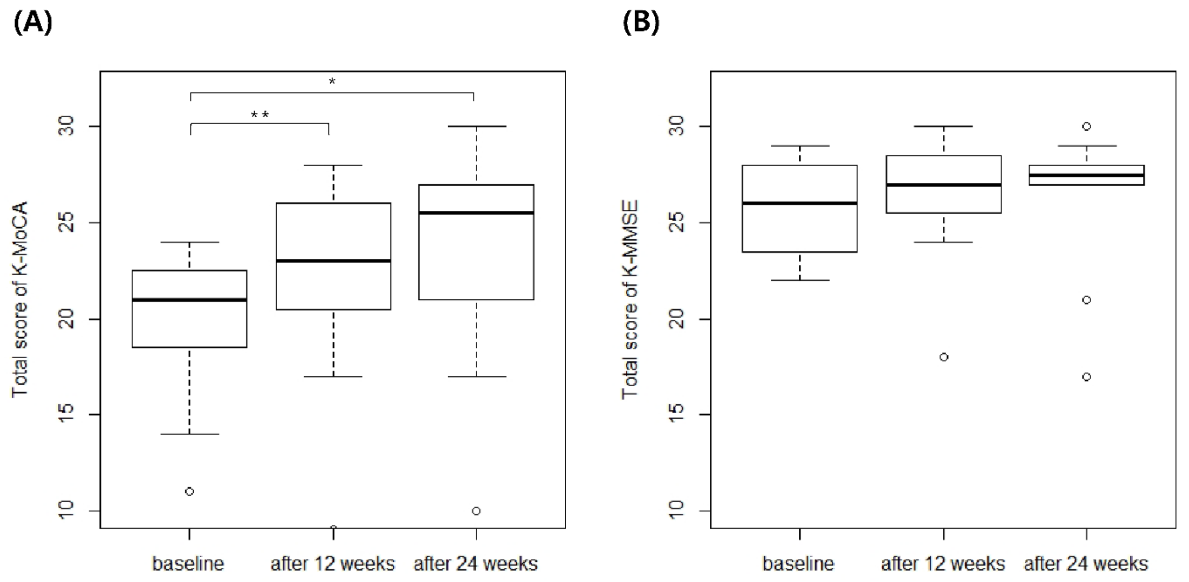

Supplementary Fig. S1. Changes in total scores of the Korean-Montreal Cognitive Assessment (K-MoCA) and the Korean-Mini Mental State Examination (K-MMSE)

(A) Total score of the K-MoCA increased after (12 and 24) weeks of treatment, compared to the baseline (*p*=0.015, *p*=0.008, respectively). (B) Total mean score of the K-MMSE tends to slightly increase after (12 and 24) weeks of treatment compared to the baseline, but it was not statistically significant (*p*=0.195, *p*=0.761, respectively). *: P<0.05 compared with the score at the baseline by the Student's paired t-test, **: P<0.001 compared with the score at the baseline by the Student's paired t-test.

Supplementary Table S1. Detailed descriptions of the Korean medicine treatment used in this study.

	Herbal formulae/dosage, acupuncture point
Herbal medicine	
Decoction (water extract)[a]	
Tonifying-Qi **formula**	
- modified Jeungsonbaekchul-san & Gakbyeongyeonsu-tang	Ginseng Radix, Achyranthis Radix, Citri Unshius Pericarpium, Crataegi Fructus, Pueraria Root, Atractylodis Rhizoma Alba, Paeoniae Radix, Poria Sclerotium, Angelicae Gigantis Radix, Agastachis Herba, Acori Graminei Rhizoma, Poria Sclerotium Cum Pini Radix 4g, Polygalae Radix, Aucklandiae Radix, Glycyrrhizae Radix et Rhizoma 2 g, Zingiberis Rhizoma Recens 6 g. 120 mL, tid.
- modified Guibi-tang (Guipi-tang, Kihi-to)	Longan Arillus, Zizyphi Semen 12 g, Zingiberis Rhizoma Recens 8 g, Angelicae Gigantis Radix, Zizyphi Fructus 6 g, Astragali Radix, Ginseng Radix, Atractylodis Rhizoma Alba, Acori Graminei Rhizoma, Massa Medicata Fermentata, Poria Sclerotium Cum Pini Radix, Crataegi Fructus 4g, Aucklandiae Radix, Polygalae Radix, Glycyrrhizae Radix et Rhizoma 2 g. 120 mL, tid.
Tonifying-Kidney **formula**	
- modified Jaeumganghwa-tang (Ziyingjianghuo-tang, Jinkoka-to)	Paeoniae Radix, Acori Graminei Rhizoma, Poria Sclerotium Cum Pini Radix, Longan Arillus 6 g, Angelicae Gigantis Radix 5g, Rehmanniae Radix Preparata, Liriopis seu Ophiopogonis Tuber, Atractylodis Rhizoma Alba, Bupleuri Radix, Massa Medicata Fermentata 4 g, Rehmanniae Radix Recens, Citri Unshius Pericarpium 3 g, Anemarrhenae Rhizoma, Phellodendri Cortex, Glycyrrhizae Radix et Rhizoma 2 g. 120 mL, tid.
- modified Yukmijihwang-hwan (Liuweidihuang-tang, Rokumijio-to)	(variation 1) Rehmanniae Radix Preparata, Longan Arillus 12 g, Corni Fructus, Dioscoreae Rhizoma 8 g, Moutan Radicis Cortex, Poria Sclerotium, Alismatis Rhizoma 6g, Acori Graminei Rhizoma, Massa Medicata Fermentata 4 g, Anemarrhenae Rhizoma, Alpiniae Oxyphyllae Fructus, Phellodendri Cortex, Polygalae Radix 2 g. 120 mL, tid.<br>(variation 2) Rehmanniae Radix Preparata 12 g, Corni Fructus, Dioscoreae Rhizoma, 8 g, Moutan Radicis Cortex, Polygalae Radix, Longan Arillus, Poria Sclerotium Cum Pini Radix, Poria Sclerotium, Alismatis Rhizoma 6 g, Atractylodis Rhizoma Alba, Acori Graminei Rhizoma, Massa Medicata Fermentata, Astragali Radix 4 g. 120 mL, tid.
- modified Gojineumja (Guzhenyinzi)	Poria Sclerotium Cum Pini Radix 8 g, Rehmanniae Radix Preparata, Acori Graminei Rhizoma 6 g, Ginseng Radix, Astragali Radix, Gardeniae Fructus, Dioscoreae Rhizoma, Angelicae Gigantis Radix, Galli Gigeriae Endothelium Corneum 4 g, Citri Unshius Pericarpium, Eucommiae Cortex, Poria Sclerotium, Glycyrrhizae Radix et Rhizoma 3 g, Atractylodis Rhizoma Alba, Corni Fructus, Phellodendri Cortex, Alismatis Rhizoma, Psoraleae Semen, Polygalae Radix 2 g. 120 mL, tid.
Calming-Liver **formula**	
- modified Ondam-tang (Wendan-tang, untan-to)	Cyperi Rhizoma, Pinelliae Tuber 10 g, Crataegi Fructus, Zingiberis Rhizoma Recens 8 g, Citri Unshius Pericarpium, Zizyphi Fructus 6 g, Phyllostachyos Caulis in Taeniam, Poria Sclerotium, Liriopis seu Ophiopogonis Tuber, Ponciri Fructus Immaturus, Ginseng Radix, Bupleuri Radix, Acori Graminei Rhizoma, Poria Sclerotium Cum Pini Radix, Massa Medicata Fermentata, Glycyrrhizae Radix et Rhizoma 4 g, Platycodonis Radix 3 g, Polygalae Radix 2 g. 120 mL, tid.

- modified Soyo-san (Jiaweixiaoyao-san, Kamishoyo-san)	Cyperi Rhizoma 10 g, Acori Graminei Rhizoma, Poria Sclerotium Cum Pini Radix, Cinnamomi Ramulus, Zingiberis Rhizoma Recens 8 g, Atractylodis Rhizoma Alba, Massa Medicata Fermentata, Paeoniae Radix, Crataegi Fructus 6 g, Poria Sclerotium, Angelicae Gigantis Radix, Curcumae Radix, Citri Unshius Pericarpium Immaturus, Bupleuri Radix, Liriopis seu Ophiopogonis Tuber, Meliae Fructus 4 g, Menthae Herba, Gardeniae Fructus, Polygalae Radix, Glycyrrhizae Radix et Rhizoma 2 g. 120 mL, tid.
- modified Daeyoung-jeon (Daying-jian, Taiei-sen)	Uncariae Ramulus cum Uncus 12 g, Rehmanniae Radix Preparata, Atractylodis Rhizoma 8 g, Cervi Cornu, Lycii Fructus, Angelicae Gigantis Radix, Eucommiae Cortex, Amomi Fructus, Achyranthis Radix, Cinnamomi Cortex, Citri Unshius Pericarpium, Pinelliae Tuber 6 g, Rehmanniae Radix, Glycyrrhizae Radix et Rhizoma, Zingiberis Rhizoma, Platycodonis Radix, Persicae Semen, Hordei Fructus Germinatus, Paeoniae Radix, Arecae Semen, Magnoliae Cortex 4 g, Angelicae Dahuricae Radix, Cnidii Rhizoma 2 g. 120 mL, bid.
Granules (manufactured)	
Tonifying-Qi **formula**	
- Bojungikgi-tang (Buzhongyiqi-tang, Hochuekki-to)	Astragali Radix, Atractylodis Rhizoma, Ginseng Radix 1 g, Angelicae Gigantis Radix 0.75 g, Bupleuri Radix, Zizyphi Fructus, Citri Unshius Pericarpium 0.5 g, Glycyrrhizae Radix et Rhizoma 0.375g, Cimicifugae Rhizoma 0.25 g, Zingiberis Rhizoma 0.125 g. 1.875 g of granule, tid.
Tonifying-Kidney **formula**	
- Uchashinki-hwan (Jishengshenqi-wan, Goshajinki-gan)	Rehmanniae Radix 1.7 g, Achyranthis Radix, Corni Fructus, Dioscoreae Rhizoma, Plantaginis Semen, Alismatis Rhizoma, Poria Sclerotium, Moutan Radicis Cortex 1.0 g, Cinnamomi Cortex, Aconiti Lateralis Radix Preparata 0.3 g. 2.5 g of granule, tid.
Calming-Liver **formula**	
- Ukgan-san (Yigan-san, Yokukan-san)	Pinelliae Tuber 1.67 g, Atractylodis Rhizoma Alba, Poria Sclerotium 1.33 g, Angelicae Gigantis Radix, Uncariae Ramulus cum Uncus, Cnidii Rhizoma, Citri Unshius Pericarpium 1.0 g, Bupleuri Radix 0.67 g, Glycyrrhizae Radix et Rhizoma 0.5 g. 3 g of granule, tid.
Acupuncture	GV20, GV24, EX-HN3, CV12, TE4, TE5, GB40, GB37, KI4 (in A hospital)<br>EX-HN1, CV12, ST22, ST25, LI4, LR3, ST36 (in B hospital)
Electroacupuncture	GV20, GV24, EX-HN3, CV12, TE4, TE5, GB40, GB37, KI4
Pharmacopuncture	placenta-pharmacopuncture on CV3
Cupping	Bladder Meridian-first line of upper back, GB21, BL23
Moxibustion	CV12

[a] Herbs added to the standard formulae are underlined.
